# Laparoscopic and open feeding jejunostomy in upper gastrointestinal pathology: a single-centre cohort study

**DOI:** 10.1007/s00464-026-12721-9

**Published:** 2026-03-30

**Authors:** Ata Ghaith, Babur Ahmed, Michael Dasa, Mohamed Alasmar, Naheed Farooq, Bilal Alkhaffaf

**Affiliations:** 1 Department of Oesophago-Gastric and Bariatric Surgery, Salford Royal Hospital, Northern Care Alliance NHS Foundation Trust, Manchester, UK; 2 Faculty of Medicine, Al-Balqa Applied University, Al-Salt, Jordan; 3 School of Medicine, New Giza University, Giza, Egypt; 4 Faculty of Biology, Medicine and Health, The University of Manchester, Manchester, UK

**Keywords:** Feeding jejunostomy, Laparoscopy, Enteral nutrition, Complications, Gastrointestinal surgery

## Abstract

**Background:**

Feeding jejunostomy (FJ) provides critical enteral access for patients undergoing treatment for upper gastrointestinal (GI) pathology. This study aimed to evaluate the safety, complication profile, and operative context of laparoscopic versus open FJ using a standardised laparoscopic technique.

**Methods:**

A retrospective cohort study included 576 patients who underwent FJ between March 2018 and August 2024 (302 laparoscopic, 274 open). Patient demographics, operative context, and postoperative complications were analysed. Outcomes are reported as frequencies with relative effect estimates and corresponding 95% confidence intervals.

**Results:**

Baseline characteristics were comparable between groups. Laparoscopic FJ was more frequently performed in the elective setting (286/302 vs. 225/274; OR 3.88, 95% CI 2.20–6.85) and during cancer staging procedures (102/302 vs. 50/274; OR 2.28, 95% CI 1.55–3.37). In contrast, laparoscopic FJ was less commonly undertaken during major resections (156/302 vs. 165/274; OR 0.71, 95% CI 0.51–0.98) and emergency surgery (16/302 vs. 49/274; OR 0.25, 95% CI 0.14–0.45). On unadjusted analysis, overall postoperative complications were more frequent following laparoscopic FJ (43/302, 14.2%) compared with open FJ (18/274, 6.5%); however, after adjustment for comorbidity burden, procedural urgency, and other clinically relevant covariates, surgical approach was not independently associated with postoperative morbidity (adjusted OR 1.25, 95% CI 0.75–2.05). Tube-related complications, including dislodgement, leakage, and small bowel obstruction, were infrequent. Lower body mass index and female sex were associated with increased odds of obstruction. Early infectious complications occurred exclusively following laparoscopic FJ (5/302, 1.7%), but this association did not persist after multivariable adjustment (adjusted OR 2.60, 95% CI 0.75–9.10).

**Conclusion:**

Laparoscopic FJ was a safe alternative to open insertion. Although unadjusted complication rates were higher following laparoscopic FJ, surgical approach was not independently associated with early morbidity after risk adjustment, supporting the use of minimally invasive techniques in appropriately selected patients.

Patients with upper gastrointestinal (GI) pathology—such as oesophageal and gastric cancer, or acute conditions including Boerhaave syndrome—frequently experience significant nutritional compromise. Dysphagia, tumour-related obstruction, systemic inflammation, and cancer-associated cachexia contribute to progressive weight loss, malnutrition, and impaired functional status. Optimisation of nutritional support in this setting is essential to improve tolerance to oncological treatment, reduce postoperative morbidity, and enhance overall outcomes [1, 2].

Feeding jejunostomy (FJ) provides reliable medium- to long-term enteral access when oral intake is inadequate or contraindicated and gastrostomy is not appropriate. It is commonly employed in patients undergoing neoadjuvant therapy, major upper GI resection, or those anticipated to require prolonged nutritional support. Compared with parenteral nutrition, FJ preserves gut integrity, reduces infectious risk, and supports immune and metabolic function [3, 4].

Traditionally, FJ has been performed via an open approach, which may be associated with increased postoperative pain, wound-related complications, and prolonged recovery [5]. Laparoscopic feeding jejunostomy was first described in 1993 [6] and has since been associated with reduced morbidity and shorter hospital stay in contemporary series [7]. However, LFJ remains technically demanding and is characterised by substantial variation in operative technique—particularly with respect to tube type, jejunal loop selection, fixation methods, and the use of subserosal tunnelling—resulting in heterogeneous outcomes and reported complication rates across studies. Furthermore, existing comparisons between laparoscopic and open FJ are limited by small sample sizes, heterogeneous patient populations, and variability in surgical context [8].

The primary objective of this study was to evaluate whether laparoscopic feeding jejunostomy, performed using a standardised technique, is associated with comparable safety and postoperative complication profiles to open feeding jejunostomy. Secondary objectives were to characterise the operative contexts in which each approach is utilised and to identify patient- and procedure-related factors associated with postoperative complications.

## Methods

### Study design and setting

This retrospective cohort study reviewed consecutive feeding jejunostomy (FJ) procedures performed between March 2018 and August 2024 at a large upper gastrointestinal tertiary referral centre serving a population of approximately 3.2 million in Greater Manchester, UK. Data were obtained from a prospectively maintained institutional database and supplemented with retrospective review of electronic medical records where required.

### Participants

All adult patients who underwent feeding jejunostomy (FJ) insertion at our institution during the study period were included. Feeding jejunostomy was performed to provide enteral nutritional access in patients with upper gastrointestinal pathology, including obstructive upper GI malignancy requiring staging laparoscopy prior to neoadjuvant therapy, major upper GI resections, and acute conditions such as Boerhaave syndrome. Both elective and non-elective (emergency) procedures were included.

As this was a retrospective observational study, no formal predefined protocol was implemented. Data were extracted from routinely documented clinical records, operative notes, and postoperative follow-up documentation.

### Data collection and variables

Patient demographic data (age, sex, body mass index [BMI]), comorbidity burden assessed using the Charlson Comorbidity Index (CCI), operative indication (malignant, benign, or acute), procedural urgency (elective vs emergency), and operative details were recorded. Upper GI pathology was categorised accordingly. Postoperative complications were documented and graded according to the Clavien–Dindo classification [9]. Follow-up and monitoring of complications was open-ended, continuing until feeding tube removal.

Variables included in subsequent multivariable analyses were selected a priori based on clinical relevance and established associations with postoperative morbidity, and included age, sex, body mass index, Charlson Comorbidity Index, underlying pathology (malignant vs benign), urgency of surgery (elective vs emergency), and surgical approach (laparoscopic vs open).

### Surgical techniques

Standardised principles guided both laparoscopic and open feeding jejunostomy insertion, with some procedural variations based on clinical context.

### Open approach

The open technique followed established principles of feeding jejunostomy formation. After standard laparotomy, the duodenojejunal flexure was identified and the first jejunal loop capable of reaching the anterior abdominal wall without tension was selected. An enterotomy was created on the antimesenteric border of the jejunum, and the feeding tube (Mic® Surgical Jejunostomy Set ENFit®, model 0301-14, Avanos Medical, Inc., Alpharetta, GA, USA) was inserted. A seromuscular Witzel tunnel was constructed using absorbable sutures, without a purse-string suture. Proximal and distal anti-torque sutures were placed to secure the jejunal loop to the abdominal wall and minimise the risk of rotation or volvulus. Tube position and patency were confirmed prior to abdominal closure.

### Laparoscopic approach

The laparoscopic technique adhered to the same core principles as the open approach, including identification of the first tension-free jejunal loop, creation of an antimesenteric enterotomy, construction of a Witzel tunnel, and placement of anti-torque sutures. Pneumoperitoneum was established at 12–15 mmHg, and the duodenojejunal flexure was identified laparoscopically. Port placement was tailored to the procedural context. When the jejunostomy was performed as a standalone procedure, ports were positioned to optimise access for feeding jejunostomy insertion. When performed as part of a concurrent operation (e.g., oesophagectomy), port configuration was adapted accordingly.

A 5-mm trocar was used to introduce the feeding tube, The Witzel tunnel was constructed using 3–0 absorbable V-Loc™ sutures. Proximal and distal anti-torque sutures secured the tube to minimise the risk of rotation or volvulus. Tube positioning and patency were confirmed laparoscopically by saline infusion and flushing.

All procedures were performed or directly supervised by consultant (attending) surgeons, with involvement of trainees where appropriate.

Jejunostomy tubes remained in place until oral intake was established, typically following adjuvant chemotherapy or recovery from upper GI surgery, and after dietitian review. Tubes were removed electively in the outpatient setting. The Mic® jejunostomy system required a local anaesthetic procedure for removal due to the presence of a Dacron cuff.

**Fig. 1:**
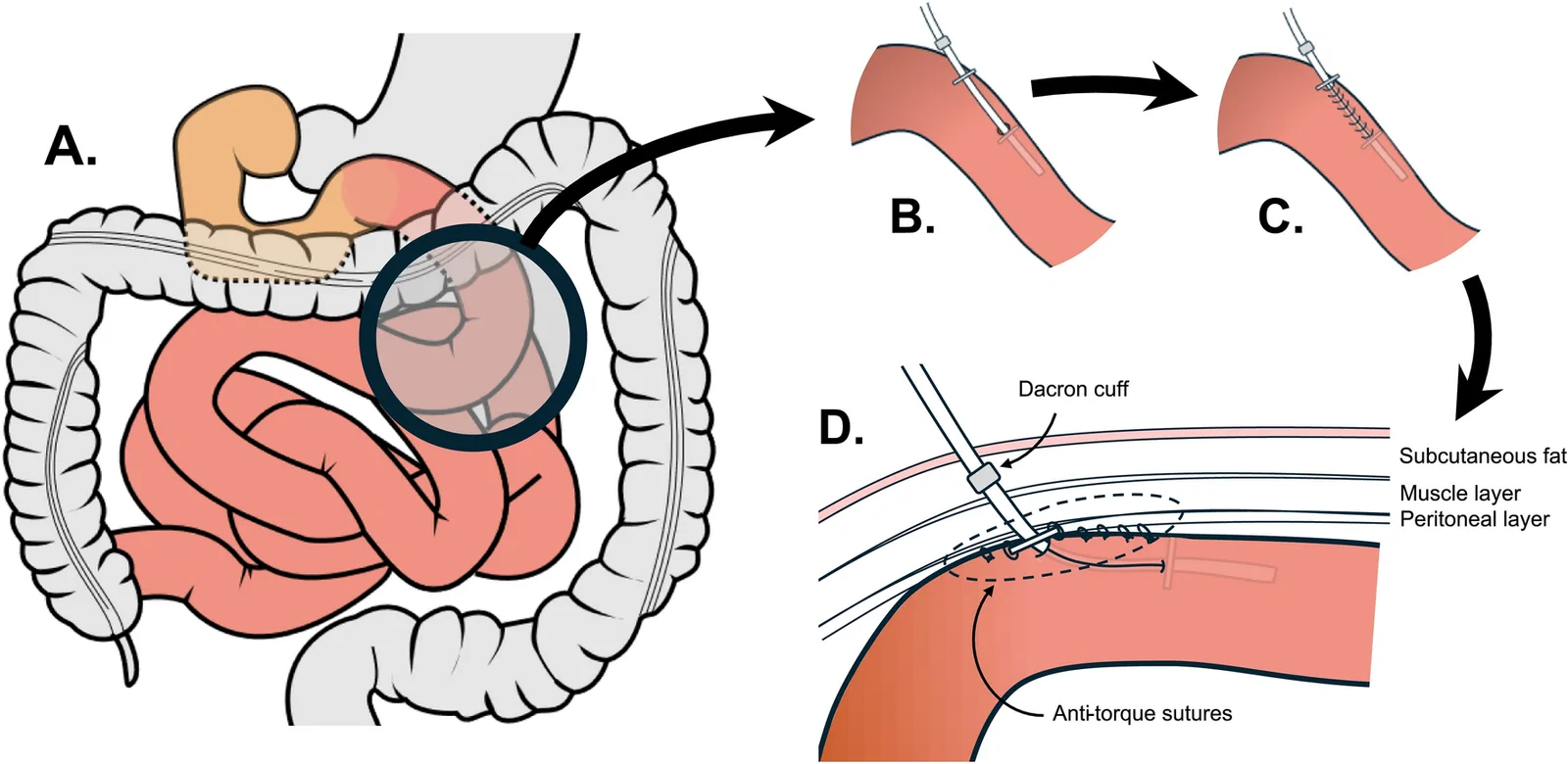
Key steps of laparoscopic feeding jejunostomy insertion using a standardized technique. **A** Selection of the first jejunal loop, **B** Insertion of tube into jejunum, **C** Witzel tunnel formation, **D** Anchoring to the anterior abdominal wall and insertion of anti-torque sutures

The key operative steps of the laparoscopic feeding jejunostomy technique are illustrated in Fig. 1.

**Fig. 2:**
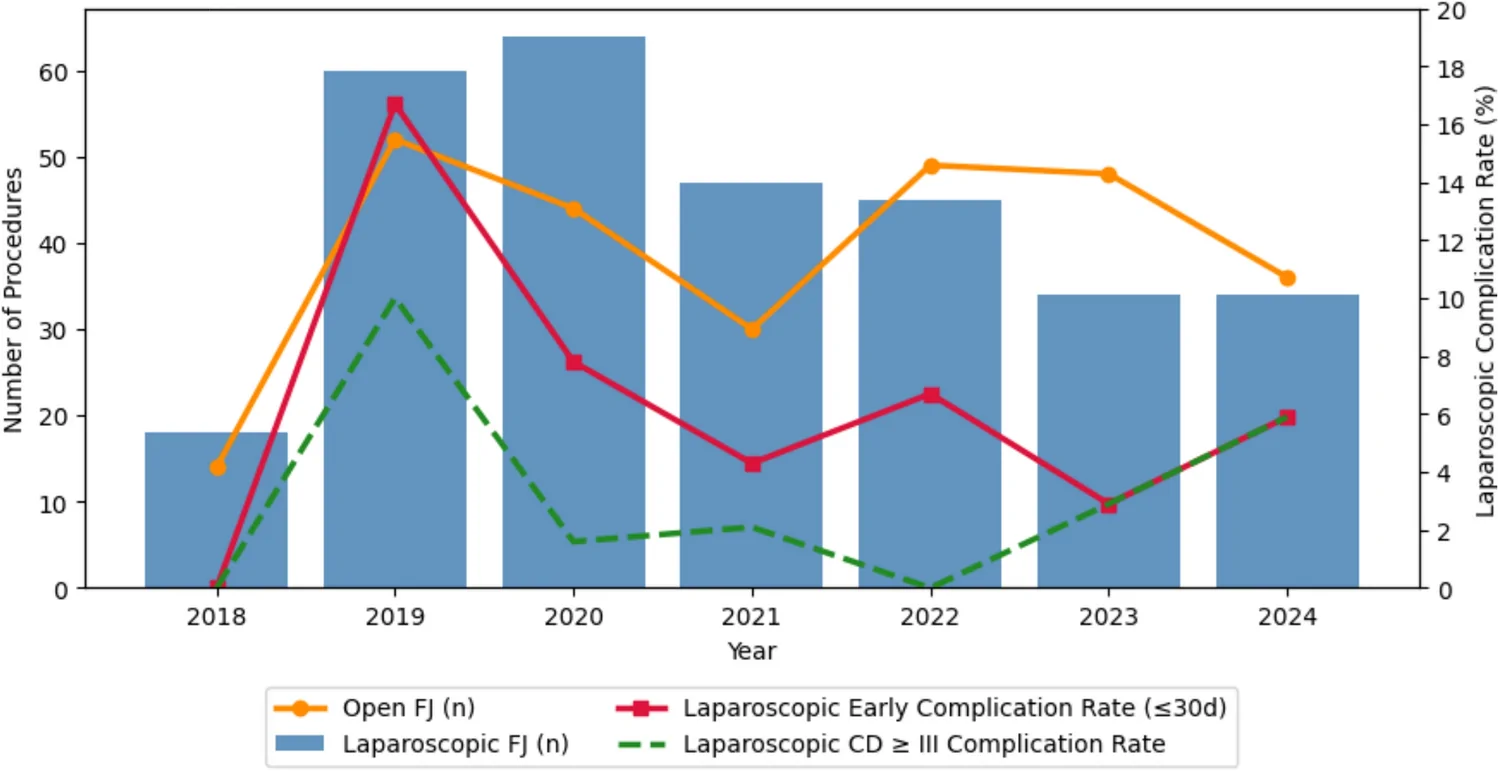
Annual open and laparoscopic feeding jejunostomy volume with laparoscopic complication rates (2018–2024). Blue bars represent the annual number of laparoscopic FJ procedures. The orange solid line indicates the annual number of open FJ procedures. The red solid line with square markers represents the laparoscopic early (≤ 30-day) complication rate, while the green dashed line represents the laparoscopic Clavien–Dindo grade ≥ III complication rate. Complication rates refer exclusively to the laparoscopic cohort (Color figure online)

### Regulatory approvals

This study was registered as a service evaluation (Registration No: 2025 166S). The study was conducted in accordance with institutional data governance policies.

### Statistical analysis

Categorical variables are presented as frequencies and percentages, and continuous variables as means with standard deviations or medians with interquartile ranges, as appropriate. Group comparisons were performed using chi-square or Fisher’s exact tests for categorical variables and Student’s *t*-tests for continuous variables, following assessment of data distribution.

Associations between surgical approach and postoperative complications were evaluated using multivariable logistic regression to adjust for potential confounding. Effect estimates are reported as odds ratios (ORs) with corresponding 95% confidence intervals. Variables were selected a priori based on clinical relevance and established associations with postoperative morbidity, and included age, sex, body mass index, Charlson Comorbidity Index, operative indication (malignant vs benign), procedural urgency (elective vs emergency), and surgical approach (laparoscopic vs open).

## Results

### Patient characteristics

A total of 576 patients underwent feeding jejunostomy (FJ) placement between March 2018 and August 2024, including 302 laparoscopic and 274 open procedures. Patient demographics and baseline characteristics are summarised in Table 1.

**Table 1:** Summary of patient and operative characteristics

	Laparoscopic *N* = 302	Open *N* = 274	Unadjusted OR^a^ (95% CI)
*Patient characteristics*			
Median age (years), (IQR)	63.0 (55–70)	64.1 (58–72)	
Median BMI (kg/m^2^), (IQR)	24.8 (21.23–27.8)	24.9 (21.83–27.4)	
Male (%)	223 (73.8%)	199 (72.6%)	1.06 (0.74–1.54)
Charlson Comorbidity Index (median), (IQR)	4 (3–5)	4(3–5)	
*Procedure type*			
Major resection (e.g. oesophagectomy)	156 (51.7%)	165 (60.2%)	0.71 (0.51–0.98)
Staging laparoscopy for cancer	102 (33.8%)	50 (18.2%)	2.28 (1.55–3.37)
Performed as primary procedure	20 (6.6%)	9 (3.3%)	2.09 (0.93–4.67)
Performed alongside other procedures (e.g. repair of Boerhaave)	24 (7.9%)	50 (18.2%)	0.39 (0.23–0.65)
Underlying pathology (malignant)	266 (88%)	222 (81%)	1.73 (1.09–2.74)
*Mode of surgery*			
Elective	286 (94.7%)	225 (82.1%)	3.89 (2.16–7.03)
Non-elective/emergent	16 (5.3%)	49 (17.9%)	

^a^Unadjusted ORs compare laparoscopic versus open feeding jejunostomy and are presented for descriptive purposes

The laparoscopic and open groups were broadly comparable with respect to age (median 63.0 years [IQR 55–70] vs. 64.1 years [IQR 58–72]), sex distribution (male 73.8% vs. 72.6%; OR 1.06), body mass index (median 24.8 kg/m^2^ [IQR 21.2–27.8] vs. 24.9 kg/m^2^ [IQR 21.8–27.4]), and comorbidity burden, with identical median Charlson Comorbidity Index scores of 4 (IQR 3–5) in both groups.

Malignant pathology accounted for the majority of feeding jejunostomy insertions in both cohorts and was more common in the laparoscopic group (266/302, 88%) than in the open group (222/274, 81%; unadjusted OR 1.73, 95% CI 1.12–2.66). Laparoscopic feeding jejunostomy was more frequently performed in the elective setting (286/302, 94.7% vs. 225/274, 82.1%; unadjusted OR 3.88, 95% CI 2.20–6.85). Conversely, emergency procedures were significantly more likely to be undertaken via an open approach (49/274, 17.9% vs. 16/302, 5.3%), corresponding to lower odds of emergency surgery in the laparoscopic group (unadjusted OR 0.25, 95% CI 0.14–0.45).

Major resections were less frequent in the laparoscopic group compared with the open group (156/302, 51.7% vs. 165/274, 60.2%; unadjusted OR 0.71, 95% CI 0.51–0.98). In contrast, laparoscopic feeding jejunostomy was more commonly performed during staging laparoscopy for malignancy, occurring in 102 of 302 cases (33.8%) compared with 50 of 274 open procedures (18.2%; unadjusted OR 2.28, 95% CI 1.55–3.37).

Feeding jejunostomy was performed as a standalone primary procedure in a minority of cases and was more frequent in the laparoscopic group than in the open group (20/302, 6.6% vs. 9/274, 3.3%; unadjusted OR 2.10, 95% CI 0.91–4.85). Conversely, feeding jejunostomy performed alongside other procedures—such as repair of Boerhaave syndrome or laparotomy for complications—was less frequent in the laparoscopic group compared with the open group (24/302, 7.9% vs. 50/274, 18.2%; unadjusted OR 0.39, 95% CI 0.23–0.65).

The median follow-up duration for feeding jejunostomy–related complications was 177 days (IQR 136–277).

### Postoperative complications

Overall complications occurred in 61 of 576 patients (10.6%) and are summarised in Table 2. On unadjusted comparison, complications were more frequent following laparoscopic feeding jejunostomy than open insertion, occurring in 43 of 302 laparoscopic cases (14.2%) compared with 18 of 274 open cases (6.5%) unadjusted OR 2.38, 95% CI 1.33–4.20.

**Table 2:** Association between surgical approach (lap vs open), over all postoperative complications and early complication: unadjusted vs adjusted ORs

Overall complications						Early complications (< 30 days)				
	Lap (%)	Open (%)	Unadjusted OR (95% CI)	Adjusted OR (lap vs open)	95% CI	Lap (%)	Open (%)	Unadjusted OR	Adjusted OR (lap vs open)	95% CI
Infection/abscess	11(3.6%)	0(0%)	–^a^	1.50	0.70–3.22	5(1.7%)	0(0%)	–^a^	2.60	0.75–9.10
Tube dislodgment	9(3.0%)	7(2.6%)	1.17 (0.43–3.19)	1.05	0.45–2.44	2(0.7%)	2(0.7%)	0.91 (0.13–6.48)	1.00	0.25–3.95
Tube leakage	7(2.3%)	4(1.5%)	1.60 (0.46–5.53)	1.20	0.48–3.02	4(1.3%)	2(0.7%)	1.83 (0.33–10.05)	1.50	0.40–5.55
Small bowel obstruction	8(2.6%)	5(1.8%)	1.46 (0.47–4.53)	1.18	0.47–2.96	8(2.6%)	4(1.5%)	1.84 (0.55–6.17	1.30	0.50–3.45
Tube fracture	3(1.0%)	2(0.7%)	1.36 (0.23–8.23)	1.10	0.27–4.38	2(0.7%)	0(0%)	–^a^	2.10	0.40–11.0
Tube blockage	5(1.7%)	0(0%)	–^a^	2.40	0.72–7.95	3(1%)	0(0%)	–^a^	2.80	0.60–13.2

Elective-only sensitivity analysis described in text

^a^Unadjusted odds ratios were not calculated for outcomes with zero events in one group. Adjusted odds ratios were estimated using multivariable logistic regression, which permits inclusion of zero-event categories through maximum likelihood–based model estimation. Adjusted models were prespecified and included age, sex, body mass index, Charlson Comorbidity Index, underlying pathology (malignant vs benign), urgency of surgery (elective vs emergency), and surgical approach (laparoscopic vs open)

Tube dislodgement was observed in 16 patients overall (2.8%), with similar frequencies following laparoscopic (9/302, 3.0%) and open feeding jejunostomy (7/274, 2.6%; unadjusted OR 1.17, 95% CI 0.43–3.19). infection complications or abscess formation were uncommon and occurred exclusively following laparoscopic feeding jejunostomy (11/302, 3.6%). Due to the absence of events in the open group, an unadjusted odds ratio could not be calculated; however, this finding suggests a crude association with surgical approach.

Tube blockage was infrequent, occurring in five laparoscopic cases (5/302, 1.7%) and in no open cases. Owing to zero events in the open group, an unadjusted odds ratio could not be calculated. Leakage rates were similarly low in both groups, occurring in seven laparoscopic cases (7/302, 2.3%) and four open cases (4/274, 1.5%; unadjusted OR 1.60, 95% CI 0.46–5.53).

Small bowel obstruction occurred in 13 patients overall (2.3%), with comparable frequencies following laparoscopic (8/302, 2.6%) and open procedures (5/274, 1.8%; unadjusted OR 1.46, 95% CI 0.47–4.53). Tube fracture was rare, occurring in five patients overall, including three following laparoscopic feeding jejunostomy (3/302, 1.0%) and two following open procedures (2/274, 0.7%; unadjusted OR 1.36, 95% CI 0.23–8.23).

Overall Clavien–Dindo grade III or higher complications occurred in 18 laparoscopic patients (18/302, 6.0%) and 12 open patients (12/274, 4.4%). On unadjusted analysis, there was no statistically significant association between surgical approach and severe morbidity (unadjusted OR 1.39, 95% CI 0.66–2.92). There were no feeding jejunostomy–related mortalities.

In a sensitivity analysis restricted to elective procedures, overall complications occurred in 33 of 286 laparoscopic cases (11.5%) compared with 13 of 225 open cases (5.8%). On unadjusted analysis, laparoscopic feeding jejunostomy was associated with higher odds of overall complications in elective cases (unadjusted OR 2.13, 95% CI 1.09–4.15). However, this analysis remains exploratory and does not account for differences in operative indication, procedural complexity, or patient-related risk factors, and should therefore be interpreted with caution.

### Subgroup analysis for early complications (≤ 30 days)

A total of 32 complications occurred within 30 days of jejunostomy insertion, including 24 events following laparoscopic procedures and 8 following open procedures. Most early complications—including tube dislodgement, leakage, blockage, fracture, and small bowel obstruction—occurred at similar frequencies between surgical approaches (Table 2).

Early infection complications were uncommon and occurred exclusively following laparoscopic feeding jejunostomy (5/302, 1.7%), demonstrating a crude association with surgical approach. However, event numbers were low. Early major complications (Clavien–Dindo grade ≥ III) occurred in 11 laparoscopic patients (3.6%) and 5 open patients (1.8%), with no statistically significant unadjusted association between surgical approach and early severe morbidity (unadjusted OR 2.03, 95% CI 0.70–5.93).

### Multivariate analysis

Multivariable logistic regression analyses were performed using variables selected a priori based on clinical relevance and established risk factors for postoperative complications (Table 3). After adjustment, surgical approach (laparoscopic vs open) was not independently associated with overall postoperative complications (adjusted OR 1.25, 95% CI 0.75–2.05). In contrast, higher comorbidity burden, as measured by the Charlson Comorbidity Index (CCI), was associated with increased odds of overall complications (adjusted OR 1.10 per point increase, 95% CI 1.00–1.20), and emergency procedures were associated with nearly two-fold higher odds compared with elective cases (adjusted OR 1.95, 95% CI 1.10–3.70).

**Table 3:** Multivariable logistic regression analysis of factors associated with postoperative complications

Over all complications			Early complication (< 30 days)		Early CD III complications	
	aOR	95% CI	aOR	95% CI	aOR	95% CI
Age (per year)	1.01	0.99–1.03	1.00	0.98–1.03	1.01	0.99–1.04
Sex: male (vs female)	1.12	0.70–1.78	1.05	0.55–2.00	1.15	0.55–2.40
BMI (per kg/m^2^)	0.97	0.91–1.03	0.96	0.89–1.04	0.97	0.90–1.04
Charlson Comorbidity Index	1.10	1.00–1.20	1.08	0.98–1.20	1.12	1.01–1.25
Urgency: emergency (vs elective)	1.95	1.10–3.70	1.95	1.05–3.80	2.05	1.05–4.00
Surgical approach: lap (vs open)	1.25	0.75–2.05	1.45	0.72–2.95	1.20	0.60–2.45
Pathology: benign (vs malignant)	1.35	0.72–2.50	1.25	0.65–2.40	1.30	0.62–2.75

Adjusted odds ratios (aORs) were derived from multivariable logistic regression models. Variables were selected a priori based on clinical relevance and known risk factors for postoperative complications and included age, sex, body mass index, Charlson Comorbidity Index, underlying pathology (malignant vs benign), urgency of surgery (elective vs emergency), and surgical approach

In analyses of specific complication types, small bowel obstruction was not independently associated with surgical approach; however, lower body mass index and female sex were significant independent predictors. Each 1 kg/m^2^ increase in BMI was associated with reduced odds of obstruction (adjusted OR 0.81, 95% CI 0.71–0.94), while female sex was associated with higher odds compared with males (adjusted OR 4.57, 95% CI 1.47–14.19).

In the early (≤ 30-day) complication subgroup, surgical approach was again not independently associated with complications (adjusted OR 1.45, 95% CI 0.72–2.95). Emergency surgery remained the principal predictor of early complications (adjusted OR 1.95, 95% CI 1.05–3.80), while comorbidity burden demonstrated an imprecise association (adjusted OR 1.08, 95% CI 0.98–1.20).

For early severe complications (Clavien–Dindo grade ≥ III), multivariable analysis identified both higher CCI (adjusted OR 1.12 per point increase, 95% CI 1.01–1.25) and emergency surgery (adjusted OR 2.05, 95% CI 1.05–4.00) as independent predictors. Surgical approach and other patient-related factors, including age, sex, body mass index, and underlying pathology, were not independently associated with early severe morbidity.

## Discussion

Feeding jejunostomy (FJ) remains a widely used method for providing medium- and long-term enteral nutrition in patients with upper gastrointestinal malignancies and complex pathologies. Over recent decades, laparoscopic techniques have gained increasing acceptance, with reported advantages including reduced postoperative pain, faster recovery, and fewer wound-related complications. However, the relative safety of laparoscopic versus open feeding jejunostomy remains debated, largely due to heterogeneous techniques, small series, and variable reporting of complications.

In this large single-centre cohort, both laparoscopic and open feeding jejunostomy were associated with low overall complication rates. Importantly, multivariable analysis demonstrated that surgical approach was not independently associated with postoperative morbidity. Instead, patient- and procedure-related factors—particularly comorbidity burden and emergency surgery—emerged as the principal determinants of adverse outcomes.

Although unadjusted analyses demonstrated higher overall complication rates following laparoscopic feeding jejunostomy, this association did not persist after risk adjustment. This discrepancy likely reflects differences in case mix and procedural context rather than an intrinsic effect of surgical approach. In the present cohort, laparoscopic feeding jejunostomy was more frequently performed in elective oncological settings and during staging or complex upper gastrointestinal procedures, which may confer a higher baseline risk of postoperative morbidity. These findings underscore the limitations of interpreting crude complication rates in isolation and emphasise the importance of multivariable modelling when comparing surgical approaches.

A sensitivity analysis restricted to elective procedures similarly demonstrated higher unadjusted complication rates following laparoscopic feeding jejunostomy, indicating that the observed crude difference is not solely attributable to emergency surgery. However, this analysis remains unadjusted and does not account for differences in operative indication, procedural complexity, or patient-related risk factors. As such, these findings further highlight the limitations of unadjusted subgroup analyses and support the use of multivariable modelling to assess independent associations.

The observed difference in overall complications on unadjusted analysis was largely driven by infectious complications or abscess formation, which occurred exclusively in the laparoscopic group. These events were infrequent in absolute terms and occurred while the jejunostomy tube was in situ. The absence of infections in the open group should be interpreted cautiously, as the small number of events limits definitive conclusions and may reflect random variation or under-reporting rather than a true protective effect of the open approach. Differences in postoperative surveillance and documentation—particularly within structured oncological care pathways where laparoscopic procedures are more commonly performed—may also have contributed to differential detection of infectious complications.

Other tube-related complications, including dislodgement, leakage, blockage, small bowel obstruction, and tube fracture, were uncommon and occurred at similar frequencies between groups. Although all infectious complications occurred in laparoscopic cases, this association did not persist after multivariable adjustment. This finding is likely multifactorial and reflects low event numbers, procedural context, and perioperative or surveillance-related factors rather than an intrinsic increase in risk associated with the laparoscopic approach. Notably, several published series have reported comparable or lower infection rates following laparoscopic feeding jejunostomy, potentially due to reduced tissue trauma and smaller incisions. [10, 11].

Following multivariable analysis, comorbidity burden—as measured by the Charlson Comorbidity Index—and procedural urgency remained the dominant predictors of postoperative morbidity. Emergency surgery was consistently associated with increased risk, reinforcing the importance of patient optimisation and careful perioperative management. These findings highlight that patient selection and procedural context, rather than surgical access alone, are central determinants of outcome following feeding jejunostomy placement.

Importantly, not all feeding jejunostomy systems are equivalent. The type of tube used can influence complication rates. In this series, the MIC (or Vygon MIC) tube was used, which has a slightly larger internal diameter and a Dacron cuff to help secure the tube in the subcutaneous tissues. These features likely contributed to the low rates of blockage and tube dislodgment. In contrast, smaller-calibre tubes such as the Freka 9-Fr feeding jejunostomy are more prone to obstruction, leakage, and accidental displacement. Such differences in equipment selection may help explain some of the heterogeneity in complication rates reported across the literature.

To contextualise the present findings, Table 4 summarises overall and infection-related complication rates reported in laparoscopic feeding jejunostomy series published over the past decade. Despite differences in study design, patient populations, and operative indications, the complication rates observed in the current study are comparable to those reported in contemporary laparoscopic series [6, 12–14]. Furthermore, our observed rates fall at the lower end of historically reported ranges for feeding jejunostomy complications described in earlier open and mixed-technique cohorts, supporting the safety of a standardised laparoscopic approach in experienced centres (tube dislodgement 3–10%, infection 1–12%) [15–19].

**Table 4:** Reported overall and infectious complication rates in laparoscopic feeding jejunostomy series published over the past decade

Study	Year	Study design	LFJ cases (n)	Main indication	Overall complications (%)	Infection/abscess (%)	Mortality (%)
Mastoridis et al. [6]	2021	Retrospective cohort	31	Oesophagogastric cancer	19.3	3.2	0
Tsai et al. [12]	2021	Retrospective cohort	29	Upper GI malignancy	0	0	0
Varshney et al. [13]	2023	Retrospective cohort	20	Upper GI malignancy	25	1	0
Komek et al. [14]	2024	Retrospective cohort	30	Upper GI pathology	20	0	3.33
Present study	2025	Retrospective cohort	302	Upper GI pathology	14.2^a^	3.6^a^	0

Data are presented as reported in the original publications. Definitions of complications varied between studies, and direct comparison should therefore be interpreted with caution. No pooled analysis or comparative statistical testing was performed

^a^Rates from the present study reflect unadjusted complication frequencies

This suggests that standardised technique, technical familiarity, and appropriate tube selection may all contribute to safe outcomes. Institutional experience appears to play a role, and is reflected in our findings of a trend of improved complication rates with time. In our centre, FJ placement is performed routinely, particularly in patients with oesophageal or complex upper GI pathology. This familiarity likely contributes to the observed low complication profile. In contrast, centres that place FJs infrequently or only in select high-risk patients may experience higher complication rates. This could stem from operator inexperience or lack of standardisation—factors that are often under-recognised but critical in procedural safety. Therefore, reported differences in morbidity may reflect these systemic variables rather than differences in the laparoscopic versus open approach per se. We also observed a clear temporal improvement in outcomes following laparoscopic feeding jejunostomy, with reductions in both early and severe complications over time. Although formal trend analysis was limited by small annual event numbers, this pattern is consistent with a procedural learning curve and increasing standardisation of technique. The low absolute number of infectious events precluded a statistically meaningful post-learning subgroup analysis, and any such comparison would have been underpowered (Fig. 2).

There remains ongoing controversy regarding the routine use of FJ during oesophagectomy. Supporters argue that it provides essential nutritional support during the postoperative phase, particularly in patients undergoing neoadjuvant chemoradiotherapy [20–22]. Opponents cite concerns regarding potential complications, particularly in cases where early oral intake is feasible. This creates a potential paradox: when jejunostomies are avoided due to concerns of complications, the few that are performed may carry increased risks due to unfamiliarity or suboptimal technique. Conversely, centres that routinely place FJs as part of upper GI cancer pathways, with consistent technique and multidisciplinary support, may see fewer complications. This observation may help reconcile apparent discrepancies in the literature.

Several limitations must be acknowledged. Firstly, this was a retrospective, single-institution analysis, subject to selection and reporting bias. The decision between open and laparoscopic technique was not randomised and may have been influenced by urgency, concurrent operations, surgeon preference, or anatomical factors. While the groups were broadly similar in baseline characteristics, variation in operative indication may reflect case complexity. Analysis of complications as a result of concurrent procedures were out of the scope of this study; the degree to which this may have influenced outcomes is unknown. In addition, the present analysis focussed on complications occurring while the feeding jejunostomy was in situ; complications arising after tube removal were not systematically captured and therefore not evaluated. long-term outcomes such as nutritional efficacy or quality-of-life impact were not assessed. Although complications were recorded contemporaneously, we acknowledge the potential for under-reporting, particularly of minor events. This may account for why no infections were identified in the open surgery group. Multivariate analysis was performed and reported, though residual confounding cannot be excluded.

## Conclusion

In this large single-centre cohort, laparoscopic and open feeding jejunostomy were both associated with low overall complication rates. Although crude complication rates were higher following laparoscopic insertion, this association did not persist after adjustment for patient comorbidity, procedural urgency, and other clinically relevant factors. Multivariable analysis demonstrated that adverse outcomes were primarily driven by patient-related and procedural factors—particularly comorbidity burden and emergency surgery—rather than surgical approach itself. Taken together, these findings support the safety of laparoscopic feeding jejunostomy when performed using a standardised technique in appropriately selected patients within experienced centre.
