# Correction: Laparoscopic and open feeding jejunostomy in upper gastrointestinal pathology: a single-centre cohort study

**DOI:** 10.1007/s00464-026-13315-1

**Published:** 2026-09-08

**Authors:** Ata Ghaith, Babur Ahmed, Michael Dasa, Mohamed Alasmar, Naheed Farooq, Bilal Alkhaffaf

**Affiliations:** 1 Department of Oesophago-Gastric and Bariatric Surgery, Salford Royal Hospital, Northern Care Alliance NHS Foundation Trust, Manchester, UK; 2 Faculty of Medicine, Al-Balqa Applied University, Al-Salt, Jordan; 3 School of Medicine, New Giza University, Giza, Egypt; 4 Faculty of Biology, Medicine and Health, The University of Manchester, Manchester, UK

**Correction to: Surgical Endoscopy (2026) 40:5607–5615**

**https://doi.org/10.1007/s00464-026-12721-9**

The original online version of this article was revised to add coauthor Michael Dasa to the list of contributors.

The original article has been corrected.

